# Health-Promoting Constituents and Selected Quality Parameters of Different Types of Kimchi: Fermented Plant Products

**DOI:** 10.1155/2021/9925344

**Published:** 2021-07-21

**Authors:** Anna Korus, Emilia Bernaś, Jarosław Korus

**Affiliations:** ^1^Department of Plant Product Technology and Nutrition Hygiene, Faculty of Food Technology, University of Agriculture in Krakow, Balicka 122 Street, 30-149 Krakow, Poland; ^2^Department of Carbohydrate Technology and Cereal Processing, Faculty of Food Technology, University of Agriculture in Krakow, Balicka 122, 30-149 Kraków, Poland

## Abstract

The aim of this study was to evaluate the quality and health-promoting constituents of several variants of kimchi obtained from Chinese cabbage, kohlrabi, white radish, and cucumbers. The level of dry matter, total soluble solids, ash, total acidity, pH, dietary fiber, and vitamins C, B_1_, and B_2_, as well as total polyphenols (TP) and antioxidant activity AA (ABTS, DPPH) in kimchi, were determined. In addition, color parameters were determined (*L*∗, *a*∗, *b*∗, *C*∗, and *h*^*o*^). Kimchi with the highest proportion of Chinese cabbage (63%) had the highest levels of dry matter (11.01 g), ash (2.57 g), and vitamins: C, B_1_, and B_2_ (51 mg, 52 *μ*g, and 242 *μ*g, respectively), expressed per 100 g of fresh weight. In addition, this product showed the highest total AA of 132.3 *μ*mol Tx/g (ABTS) and 49.7 *μ*mol Tx/g (DPPH) due to its high level of TP (194 mg/100 g). Cucumber-derived kimchi (85%) also had a high content of TP (147 mg/100 g) and high AA of 88.7 *μ*mol Tx/g (ABTS) and 36.3 *μ*mol Tx/g (DPPH). Additionally, stuffed kimchi from kohlrabi (88%) had the highest amounts of total dietary fiber, 3.65 g/100 g fresh weight. In all products, red (*a*∗) and yellow (*b*∗) were the dominant colors, with values of *L*∗ ranging between 32.63 and 53.16. In general, our studies have shown that depending on the raw materials used, kimchi is a good source of dietary fiber but also vitamins and polyphenols.

## 1. Introduction

As the popularity of exotic cuisines increases, the dietary preferences of the population are changing. Products traditionally consumed in Asia can be recommended as a novel alternative in some cultures and as an important part of a healthy diet. For example, in the diet of many European countries, which is a prototype of the modern nutritional pattern, large amounts of red meat, dairy products, refined grains, and sugar are mainly consumed [1]. Such a diet causes fundamental changes in the human body, including increased production of reactive oxygen species (ROS) and oxidative stress, as well as the development of hyperinsulinemia and insulin resistance [2]. This causes a rapid increase in civilization diseases such as obesity, diabetes, cardiovascular and autoimmune diseases, cancer, and Alzheimer's disease, which are rare or virtually nonexistent among other non-Western populations [2]. This is often associated with variations in diet. A proper diet can effectively prevent many diseases and also alleviate symptoms of COVID-19, which are highly related to the aforementioned underlying diseases, and is particularly important under current circumstances [3]. The consumption of fermented food affects the condition of the gut microbiota, while gut dysfunction may influence the severity of COVID-19 [4–6].

Fermented foods have unique prohealth properties, primarily due to the presence of functional microorganisms with probiotic, antibacterial, and antioxidant properties [7]. Probiotic properties are mainly demonstrated by bacteria belonging to the group of so-called lactic acid bacteria (LAB), primarily *Lactobacillus*, *Pediococcus*, *Enterococcus*, and *Leuconostoc* [8, 9]. These bacteria improve the human intestinal microbial balance and enhance health by inhibiting the growth of pathogens such as *Escherichia coli*, *Salmonella*, and *Staphylococcus* [10]. *Lactobacillus* bacteria have the Generally Recognized as Safe (GRAS) status, which indicates that they are safe, and thus, lactic acid (LA) fermentation is common in many branches of the food industry [10, 11]. Among the microorganisms responsible for fermentation, *Lactobacillus* are important in terms of the sensory and qualitative characteristics of the product. In addition, fermentation extends the shelf-life and increases the safety of fermented products [8].

LA fermentation plays an important role in vegetable preservation. Kimchi is a product obtained from fermented vegetables with high prohealth properties, associated with traditional fermented food in Korea prepared and consumed since the 3^rd^ and 4^th^ century AD [12]. However, it is also often consumed in other East Asian countries such as Japan and China [13]. Currently, there are more than 167 types of kimchi produced, depending on the region and the main component, including cruciferous vegetables (Chinese cabbage, radish, and kohlrabi), containing a number of chemical compounds with strong health-promoting potential. Kimchi is a low-calorie food because its calorific value is below 20 kcal/100 g. Furthermore, it is an excellent source of vitamin C, B-group vitamins, and minerals such as sodium, calcium, potassium, iron, and phosphorus, as well as dietary fiber content of up to 4 g/100 g of the product, depending on kimchi variant [14, 15]. Kimchi is considered a plant-based probiotic product with health benefits, as it exhibits antiatherosclerotic and anticancer properties, as well as positively influencing the treatment of diseases associated with overweight and obesity [12, 16, 17]. Additionally, it is recognized as one of the top five healthiest foods in the world [7, 12, 18].

Chinese cabbage (*Brassica rapa* var. *pekinensis*) is usually the main ingredient of kimchi. However, many other types of vegetables can also be used to prepare kimchi, as well as many different spices. Their type certainly affects the level of quality parameters of the product and many components, e.g., dietary fiber and vitamins. Nevertheless, according to our best knowledge, there is a lack of information related to the differences in, e.g., health-promoting properties or color between kimchi products from different raw materials. Therefore, the aim of this study was to evaluate selected quality parameters, including color and health-promoting constituents of several kimchi variants obtained from Chinese cabbage, kohlrabi, white radish, and cucumbers. The products were evaluated immediately after LA fermentation when they obtained a pH of 3.5.

## 2. Materials and Methods

### 2.1. Material

The experimental material consisted of several variants of kimchi, all of which were obtained by lactic fermentation of selected plant raw materials. The products were manufactured at the Department of Plant Product Technology and Nutrition Hygiene in Krakow (Poland). The following kimchi variants were prepared from

1. Chinese cabbage
2. White radish
3. Chinese cabbage and pears (white kimchi)
4. Kohlrabi
5. Ground cucumbers
6. Stuffed Chinese cabbage in brine

### 2.2. Production of Kimchi

Depending on the variant, the following basic raw materials and ingredients were used in the production of kimchi:

1. Chinese cabbage (63%), carrots (12%), apples (8%), light soy sauce (6%), ginger (4%), garlic (2%), and green part of scallions without bulb (1.5%), gochugaru (1.5%), sugar (1%), and salt (1%)
2. Japanese white radish (86%) and soy sauce (5%), chives (3%), ginger (2%), sugar (1.5%), garlic (1%), salt (1%), and gochugaru (0.5%)
3. Chinese cabbage (52%), pears (42%), and ginger (2%), garlic (1.5%), chives (1%), salt (1%), and gochugaru (0.5%)
4. Kohlrabi (90%) and ginger (2%), soy sauce (3%), garlic (1%), chives (1%), sugar (1%), salt (1%), and gochugaru (1%)
5. Ground cucumbers with peel (85%), chives (4%), spring onion (4%), garlic (2.5%), sugar (2%), gochugaru (1.5%), and salt (1%)
6. Chinese cabbage (79%); filling components: white radish (11%), carrot (6%), raw red pepper (2%), chives (1%), and pine nuts (1%); and brine components: water (50%), pears (28%), apples (15%), onion (6%), ground garlic (0.3%), ground ginger (0.2%), and salt (0.5%)

Fresh vegetables, fruits, and other ingredients from organic farming purchased from the local supermarkets (Kraków, Poland) were used for fermentation. Raw materials (vegetables, fruits, and spices) from organic farming were of high quality, i.e., fresh, undamaged by disease or pests. The production process included pretreatment of raw materials and fermentation. Preliminary processing, depending on kimchi variant, included the following: sorting the material; washing; removing the outer leaves of Chinese cabbage; peeling (kohlrabi, radish, carrots, onions, apples, and pears); cutting; and mixing ingredients according to the recipe for a given variant. The resulting mixture was then tightly packed into glass jars. The surface was protected with foil and weight-loaded. The surface was protected with foil and loaded with a weight equal to 10% of the weight of the raw materials in the jar. Then, the product was allowed to spontaneous fermentation. In the case of stuffed kimchi, the cabbage was cut lengthwise into four parts. Each layer of leaves was filled with the previously prepared filling and covered with brine. In the initial stage, LA fermentation was carried out at 20°C ± 2°C (2 days) and then at 15°C ± 2°C (10 days), until pH 3.5 was obtained. The products were evaluated immediately after the end of the fermentation process.

### 2.3. Preparation of Material for Analysis

After fermentation, each type of kimchi was thoroughly mixed and samples were taken for analysis. To determine dry matter, total soluble solids, ash, total acidity, pH, dietary fiber, and vitamins B_1_ and B_2_, each type of kimchi was homogenized with distilled water (1 : 1). The sample for the determination of vitamin C was homogenized (1 : 1) with oxalic acid solution (2%). Dietary fiber and vitamins B_1_ and B_2_ were determined in the lyophilized material. For this purpose, a portion of kimchi previously homogenized with water was frozen at -40°C in a blast freezer (Feutron GmbH, model number 3626-51), then freeze-dried (CHRIST Gamma 1-16 LCS Plus, GmbH). Color parameters were determined in kimchi before homogenization, after mixing the sample.

### 2.4. Chemical Analysis of Kimchi

The levels of basic product parameters (dry matter, total soluble solids, ash, total acidity, pH, and dietary fiber) were determined by AOAC methods [19]. Dry matter content was determined by oven drying the sample at 105°C in a laboratory dryer (POL-EKO APARATURA, Wodzisław Śląski, Poland); total soluble solids using a refractometry method (Abbe KRŰSS AR4, Germany), at a scale of 0-95%; and ash content by incineration of a sample at 460°C in L9/S 27 furnace oven (Nabertherm GmbH, Lilienthal, Germany). Active acidity (pH) was measured using a potentiometric method (pH-/Ion Meter 692, Switzerland). The total acidity was determined using standard titration against 0.4% (*w*/*v*) NaOH and calculated as LA. Dietary fiber content—soluble and insoluble fractions—and the total was determined using the enzymatic-gravimetric method [20].

### 2.5. Vitamin Analysis

The vitamin C content was determined as the sum of ascorbic acid (AA) and dehydroascorbic acid (DHAA) using the spectrophotometrical method [21]. Oxalic acid solution (2%) was used for the extraction of the ascorbic acid. After quantitative reduction of 2,6-dichlorophenolindophenol dyestuff by ascorbic acid and extraction of the excess dyestuff using xylene, the excess was measured at 500 nm.

Vitamin B_1_ (thiamine) and B_2_ (riboflavin) content was determined using HPLC [22, 23] in the lyophilized material. Thiamine and riboflavin were determined simultaneously using a liquid chromatography with a fluorescence detector. An HPLC chromatograph (Merck Hitachi) equipped with L-7612 on-line degasser, L-7250 Programmable Autosampler, L-7100 pump, L-7480 fluorescent detector, L-7360 thermostat Oven Columns (Merck), and D-7000 Interface, with D-7000 HPLC System Manager (HSM) software, was used for the detection of vitamins. The analysis was performed on a Bionacom Velocity C18 PLMX column (4.6 mm × 250 mm, 5 *μ*m) obtained from Bionacom Ltd. (UK) together with the precolumn from the same company. The measurement was made at the excitation and emission wavelengths of 360/503 nm. The mobile phase used was a mixture of water and acetonitrile. Gradient elution was performed as follows: *t* = 0, water/acetonitrile ratio of 88 : 12, and *t* = 12, water/acetonitrile ratio of 0 : 100 at 22°C with a flow rate of 0.9 mL/min. The external standards of thiamine and riboflavin were used for the identification of these compounds and their quantitative analysis, respectively, in hydrochloric and acetic acid. The concentration of standards was in range: 0.02-2.00 *μ*g/mL of solution for vitamin B_1_ and 0.02-1.00 *μ*g/mL of solution for vitamin B_2_; correlation coefficient (*R*^2^) of the calibration graphs was 0.988 for vitamin B_1_ and 0.997 for vitamin B_2_. Limit of detection (LOD) was >0.001 *μ*g/mL of sample for vitamin B_1_ and >0.003 *μ*g/mL of sample for vitamin B_2_, while limit of quantification (LOQ) was >0.004 *μ*g/mL and >0.010 *μ*g/mL, respectively.

### 2.6. Polyphenol Content and Antioxidant Activity

Determination of polyphenols and antioxidant activity was achieved via extraction of the sample with 80% methanol acidified with HCl (0.5%). Total polyphenol content was determined by a spectrophotometric method with Folin-Ciocalteu reagent [24]. The total flavonoid content was detected spectrophotometrically based on *flavonoid-aluminum chloride* (AlCl_3_) complexation [25]. Flavonols and phenolic acids content were measured spectrophotometrically, according to the procedure described by Oomah et al. [26].

Antioxidant activity was determined via two spectrophotometric methods: scavenging activity against DPPH (1,1-diphenyl-2-picrylhydrazyl) free radical [27], and application of ABTS (2,2′-azinobis(3-ethylbenzthiazoline-6-sulfonate)) cation radical [28]. For the above methods, absorbance was measured at 516 nm and 734 nm, respectively. The results were expressed as Trolox equivalent antioxidant activity in *μ*mol Tx/g. Trolox was prepared in phosphate-buffered saline (PBS) (2.5 mM/L; pH 7.4).

Vitamin C, polyphenols, and antioxidant activity content were determined using Hitachi U-2900 double beam spectrophotometer (Hitachi Europe Ltd.).

### 2.7. Color of Kimchi

The color assessment was performed via an instrumental method using CIE (*L*∗*a*∗*b*∗) system [29]. Measurements were conducted using the reflection method with Konica Minolta CM-3500d (Konica Minolta, Inc., Japan) with reference to illuminant D65 and a visual angle of 10^o^. The following parameters were measured: *L*∗ (lightness) (*L*∗ = 0 blackness, *L*∗ = 100 whiteness), *a*∗ (the proportion of green) (*a*∗<0) or red (*a*∗>0), *b*∗ (the proportion of blue) (*b*∗<0) or yellow (*b*∗>0), *C*∗ (chroma), and *h*^*o*^ (hue angle).

### 2.8. Statistical Analyses

The results obtained, expressed in fresh weight, were statistically analyzed using one-way analysis of variance (ANOVA) on the basis of the Snedecor *F* and Student's *t*-tests. The least significant difference (LSD) was calculated at the probability level of *p* < 0.05. Statistica 12.0 program was applied (StatSoft; US).

## 3. Results and Discussion

### 3.1. Basic Chemical Composition

There were significant differences in individual parameters, due to the variety of raw materials (Table 1). It was determined that the investigated products contained the following: dry matter content ranged from 5.80 g/100 g (D) to 11.01 g/100 g (A) and most of the dry matter which was the total soluble solids (3.80-9.80%). Ash is an important component of kimchi dry matter. Tamang et al. [30] reported that the ash content of white and stuffed kimchi is 1.5 g/100 g and 2.8 g/100 g fresh weight, respectively, which is in agreement with our results. From a nutritional point of view, it should be emphasized that micronutrient availability in fermented vegetables increases due to significant reduction phytic acid by LAB fermentation [31].

During fermentation organic acid content increase with decreasing pH value, which affects the sensory features of kimchi [32]. It is known that a lower pH value reduces product spoilage [9], in which pH below 4.2 ensures microbiological stability of the product. In the case of kimchi products, reduction of pH to an average of 3.44 was accompanied by LA production to an average of 1.58 g/100 g (Table 1). The breakdown of carbohydrates in the fermented raw material leads to the formation of many organic acids, which are responsible for the characteristic and specific flavor of kimchi. Therefore, the pH value is a basic indicator for the determination of the appropriate quality of kimchi [33].

Nowadays, dietary fiber is considered an essential aspect of a healthy diet, especially in the prevention of obesity, which is an epidemic disease worldwide [34]. The effect of obesity in the development of many serious diseases is well documented, e.g., cardiovascular diseases, dysglycemia, metabolic syndrome and type 2 diabetes mellitus, elevated blood pressure, and hypertension [35]. Recent reports have shown that obese patients also exhibit more severe symptoms with COVID-19 infection, resulting in respiratory failure, need for mechanical ventilation, and higher mortality [36]. Hence, there have been considerable interests of both nutritionists and consumers to include fiber-containing products in daily diet. Besides high amounts of minerals, vitamins, and other health-promoting compounds, kimchi has high levels of dietary fiber [17, 37]. According to the EU regulation [38], products containing at least 3 g of fiber per 100 g (or 1.5 g per 100 kcal) may be labeled as a “source of fiber,” and a product containing 6 g of fiber per 100 g (or 3 g of fiber per 100 kcal) may be labeled “high fiber.” Herein, the analyzed products' total dietary fiber content ranged from 1.65 g/100 g (C) to 3.80 g/100 g fresh weight (F), making kimchi a high-fiber product due to its low calorific value (below 20 kcal/100 g). Most dietary fiber was an insoluble fraction (64-78%), except for kohlrabi kimchi (D), in which the soluble fraction was dominant (69%) (Table 2).

Dietary recommendations for the intake of individual nutrients vary from country to country. Based on the recommendations developed for the Polish population (20-25 g/day for adults), the content of total fiber in 100 g of the investigated kimchi variants varied from 7 to 17% of the average Adequate Intake (AI) [39]. In turn, based on the U.S. Department of Health and Human Services recommendations for soluble fiber intake (5-10 g/day), 100 g of the tested kimchi variants covered 5-34% of the average AI [39]. Thus, it can be seen how important that from a nutritional point of view it is to choose the right products from among similar ones.

### 3.2. Vitamins

Although numerous food products contain essential vitamins, deficiencies of these compounds still exist in many countries, due to an unbalanced diet. B vitamins are involved in metabolic processes, mainly as coenzymes, and vitamin C increases the absorption of iron, boost the immune system, and participates in many physiological functions [40]. Vitamin C as well as B vitamins can be easily leached or destroyed during cooking and food processing [41, 42]. Therefore, the introduction of fermented, nonheat-treated products in a diet can have substantial advantages. Such fermented products are a valuable source of vitamin C, in which its strongly acidic medium protects the products from oxidation. Content of vitamin C in various foods is relatively high (10–100 mg/100 g), and in some cases, it reaches units of grams per 100 g. This is possibly related to the fact that vitamin C is formed from sugars, which are common compounds in different organisms [43]. Peñas et al. [44] state that the content of vitamin C in fermented cabbage (sauerkraut) is even higher than in most fresh vegetables.

Analysis of kimchi showed significant levels of vitamin C content, which ranged from 19.40 to 50.64 mg/100 g fresh weight, where the highest content was recorded for Chinese cabbage (A) (Table 3). The authors' opinions on the vitamin C content of fermented vegetables are different. According to Zhao et al. [45], the content of this vitamin in fermented Chinese cabbage was 43.2 mg/kg, while the value noted by Peñas et al. [46] in sauerkraut was 20.1 mg/100 g. On the other hand, Martinez-Villaluenga et al. [47] showed that vitamin C content of sauerkraut was ranged from 156.72 mg/100 g d.w. to 256.31 mg/100 g d.w. The highest concentration was observed in low-sodium (0.5% NaCl) sauerkraut. Özer and Kalkan Yıldırım [48] noted 77.42 mg ascorbic acid/100 g d.w. in kimchi from Chinese cabbage. Martinez-Villaluenga et al. [47] report that vitamin C content is affected by chemical and enzymatic oxidation. Therefore, cutting or shredding can affect the vitamin C content. For example, excessive trimming of leafy vegetables leads to loss of outer leaves containing more vitamins than inner leaves. The increase in vitamin C levels in fermented vegetables may be influenced by the type of added ingredients used in fermentation [49]. Wojdyła and Wichrowska [50] showed that the addition of beet, carrot, and apple to cabbage fermentation significantly increased the vitamin C content of sauerkraut by 3.5, 1.2, and 0.6%, respectively.

Relating the values presented in Table 3 to the Recommended Dietary Allowance (RDA), 100 g of kimchi variants covers 22-56% of the RDA for men (90 mg/day) and 26-68% of the RDA for women (75 mg/day) [39].

B vitamins are found in many foods. However, they are water-soluble, and many of them are temperature sensitive, which contributes to significant losses during processing. Because of this as well as poor diet, B vitamin deficiency is common in humans. Plant-based fermented foods can supplement the diet with nutrients, including vitamins. Numerous reports have detailed the health benefits attributed to kimchi due to the presence of B-group vitamins [12, 15, 49]. The level of vitamins B_1_ and B_2_ in the kimchi variants are presented in Table 3. Vitamin B_1_ and B_2_ content was determined as 11-52 *μ*g/100 g and 29-242 *μ*g/100 g fresh weight, respectively. This content covers 1-4% of the RDA for vitamin B_1_ and 2-19% of the RDA for vitamin B_2_ for men, as well as 1-5% and 3-33% of the RDA for women, respectively [39]. It is worth noting that Chinese cabbage kimchi (A) had the highest content of both vitamins, while white radish kimchi had the statistically lowest content. Certain strains of lactic acid bacteria (LAB) have the capability to synthesize water-soluble vitamins such as those included in the B-group [51]. For example, increased levels of B-group vitamins have been reported in fermented soybeans (tempeh) and sauerkraut [52, 53]. According to Russo et al. [54], B vitamins are synthesized from various nonvitamin precursors by certain bacteria in plant fermented foods.

### 3.3. Polyphenols and Antioxidant Activity

The product's functional features are increasingly desired by consumers, in which an important role is assigned to antioxidants in vegetables [55, 56]. Plant-based food products are known to protect against degenerative diseases and aging due to their antioxidant properties, mainly resulting from their high polyphenol content [57].  Table 4 indicates that Chinese cabbage kimchi (A) had the highest level of total polyphenols (193.7 mg/100 g). High levels of total polyphenols were also noted in kimchi from cucumber (E), white radish (B), and Chinese cabbage with pears (C). Özer and Kalkan Yıldırım [48] showed that fermented cabbage products, including pickled cabbage, sauerkraut, and kimchi, have a high content of polyphenols compounds, considerable antioxidant properties, and, therefore, beneficial influence toward organism.

According to Melini et al. [58], fermentation of plant products increases their antioxidant activity. Hence, through the consumption of products containing such biologically active substances, antioxidants can be provided to our daily diet [59]. In this study, fermented products showed ABTS and DPPH radical-scavenging activities in the range of 50.6-132.3 *μ*mol Tx/g and 18.7-49.7 *μ*mol Tx/g, respectively (Table 4). The highest level of antioxidant activity was observed in Chinese cabbage product (A) (132.3 and 49.7 *μ*mol Tx/g for ABTS and DPPH, respectively). Relatively high activity was determined from kimchi made of cucumbers (E) (88.7 *μ*mol Tx/g (ABTS) and 36.3 *μ*mol Tx/g (DPPH)). Özer and Kalkan Yıldırım [48] claimed that fermented foods such as sauerkraut, kimchi, and pickled cabbage can be considered high-antioxidant dietary food. Epidemiological and clinical studies conducted by Pounis et al. [60] proved that vitamins with antioxidant effects and flavonoids occurring in selected food products influenced the reduction of oxidative stress.

One of the most important groups of polyphenolic compounds is flavonoids, as they are considered health-promoting dietary supplements and prevent civilization diseases. Current reports have described flavonoids as essential components for many nutraceutical, pharmaceutical, therapeutic, and cosmetic applications [61]. Flavonoid content in kimchi, depending on the variant, was 20.94-55.20 mg of catechin/100 g, and the highest was in Chinese cabbage kimchi (A) (Table 5). In this antioxidant group, flavonol content was 9.55-24.21 mg of quercetin/100 g, in which kimchi made of cucumbers (E) and Chinese cabbage (A) was the best source. In addition, kimchi obtained from Chinese cabbage (A) and cucumbers (E) showed the highest level of phenolic acids (22.82 and 20.86 mg of caffeic acid/100 g, respectively) (Table 5). Seong et al. [62] found four phenolic acids (caffeic acid, sinapic acid, *p*-coumaric acid, and ferulic acid) and one flavonoid (myricetin) in Chinese cabbage. The outer leaves contained the most of these compounds, both in terms of quantity and composition (e.g., caffeic acid and ferulic acid were present only in outer leaves). The cited authors conclude that the outer leaves are the most valuable from a nutritional point of view and can help to improve the health value of products.

### 3.4. Instrumental Color Analysis

The color of the food is commonly associated with product quality. This is the feature on the basis of which the consumer evaluates the product and selects it [63]. Consumers first assess products visually since they very rarely have an opportunity to taste a particular product. There were significant differences between the products in the assessed color parameters (Table 6). Kimchi variant (C) exhibited the highest lightness (*L*∗ = 53.16), but the lowest value of *a*∗ (6.35). In turn, variants ((B) (*L*∗ = 32.63) and (E) (*L*∗ = 33.93)) were the darkest. Moreover, the distinguishing feature of kimchi from cucumbers (E) was its high share of redness (*a*∗ = 12.10), due to significant gochugaru content (2%). Red pepper powder (*Capsicum annuum* L.), known as gochugaru, is the main seasoning responsible for the spicy flavor and attractive kimchi color. The presence of gochugaru affects kimchi fermentation by increasing the concentration of secondary metabolites during the process [64]. Additionally, red pepper powder is a crucial factor that influences the ontogeny of *Weissella cibaria* during kimchi fermentation. Kang et al. [64] reported that *W. cibaria* possesses anti-inflammatory, anticancer, and antibacterial properties.

The share of yellow (*b*∗) in kimchi had an average level of 24.79, and the largest was recorded in kimchi variants ((A) (*b*∗ = 28.62) and (E) (*b*∗ = 27.20)). In both products, color saturation (*C*∗) was higher compared to other kimchi variants. This parameter is a measure of color intensity, and the higher the value, the more intense and vivid the color. The values determined for parameter *h*^*o*^ were in the range of yellow (62.93-74.51).

## 4. Conclusions

Nowadays, consumers are becoming increasingly aware and interested in foods with high nutritional value, especially their health-promoting effects on the human body. Consumers, thanks in part to available media, gain greater knowledge and awareness of the impact of a healthy lifestyle on the human body. Therefore, modern consumers are searching for high-quality food, which undergoes as little processing as possible. Consumption of food produced only from natural components, without added artificial colors or preservatives, has a considerable beneficial impact on the body's overall condition. Fermented products meet the above consumer expectations, as they are stable, do not require the addition of preservatives and, above all, have high nutritive value. Therefore, kimchi deserves great interest. As presented in this paper, the wide range of raw materials for kimchi production, especially Chinese cabbage, makes it possible to obtain functional products with high-antioxidant properties. Kimchi can be recommended for consumption as a source of dietary fiber, ash, vitamin C and B, and high amounts of phenolic content. Therefore, consumption of such products with a substantial amount of health-promoting compounds can contribute to the improvement of well-being, condition, and overall health.

## Figures and Tables

**Table 1:** Dry matter, total soluble solids, ash and acidity of selected types of kimchi.

Type of kimchi^a^	Dry matter (g/100 g)	Total soluble solids (^o^Bx)	Ash (g/100 g)	pH	Total acidity (g lactic acid/100 g)
A	11.01 ± 0.52^e^	9.80 ± 0.10^d^	2.57 ± 0.09^d^	3.53 ± 0.02^c^	1.42 ± 0.07^a^
B	7.11 ± 0.17^c^	6.40 ± 0.12^c^	1.40 ± 0.16^b^	3.43 ± 0.01^b^	1.62 ± 0.05^b^
C	7.57 ± 0.24^c^	6.30 ± 0.11^c^	1.47 ± 0.04^b^	3.33 ± 0.01^a^	1.76 ± 0.05^c^
D	5.80 ± 0.15^a^	4.13 ± 0.09^b^	0.69 ± 0.03^a^	3.44 ± 0.01^b^	1.62 ± 0.02^b^
E	8.44 ± 0.20^d^	6.30 ± 0.11^c^	1.49 ± 0.02^b^	3.59 ± 0.01^c^	1.40 ± 0.05^a^
F	6.17 ± 0.30^b^	3.80 ± 0.08^a^	1.92 ± 0.06^c^	3.37 ± 0.01^a^	1.67 ± 0.04^b^

Values are presented as the mean value ± SD (*n* = 4), in fresh matter. Different letters in the same columns indicate significant differences (*p* < 0.05). ^a^Type of kimchi—A: from Chinese cabbage; B: from white radish; C: from Chinese cabbage and pears (white kimchi); D: from kohlrabi; E: from ground cucumbers; F: stuffed Chinese cabbage in brine (ingredients: see Materials and Methods).

**Table 2:** Content of dietary fiber of selected types of kimchi, g/100 g.

Type of kimchi^a^	Dietary fiber		
	Insoluble	Soluble	Total
A	1.67 ± 0.22^b^	0.48 ± 0.05^b^	2.15 ± 0.11^b^
B	1.27 ± 0.15^a^	0.71 ± 0.04^c^	1.98 ± 0.09^a^
C	1.24 ± 0.31^a^	0.41 ± 0.05^a^	1.65 ± 0.08^a^
D	1.12 ± 0.21^a^	2.53 ± 0.16^e^	3.65 ± 0.61^c^
E	1.77 ± 0.11^b^	0.73 ± 0.03^c^	2.50 ± 0.19^b^
F	2.83 ± 0.33^c^	0.97 ± 0.06^d^	3.80 ± 0.44^c^

Values are presented as the mean value ± SD (*n* = 4), in fresh matter. Different letters in the same columns indicate significant differences (*p* < 0.05). ^a^Type of kimchi—A: from Chinese cabbage; B: from white radish; C: from Chinese cabbage and pears (white kimchi); D: from kohlrabi; E: from ground cucumbers; F: stuffed Chinese cabbage in brine (ingredients: see Materials and Methods).

**Table 3:** Vitamin content of selected types of kimchi.

Type of kimchi^a^	Vitamin C (mg/100 g)	Vitamin B_1_ (*μ*g/100 g)	Vitamin B_2_ (*μ*g/100 g)
A	50.64 ± 1.62^e^	52 ± 1^d^	242 ± 0^f^
B	36.36 ± 0.54^c^	11 ± 1^a^	29 ± 1^a^
C	19.40 ± 1.31^a^	16 ± 2^b^	79 ± 2^d^
D	38.58 ± 1.32^d^	15 ± 1^b^	47 ± 1^b^
E	20.65 ± 0.59^a^	26 ± 1^c^	86 ± 3^e^
F	25.49 ± 0.45^b^	17 ± 3^b^	53 ± 1^c^

Values are presented as the mean value ± SD (*n* = 4), in fresh matter. Different letters in the same columns indicate significant differences (*p* < 0.05). ^a^Type of kimchi—A: from Chinese cabbage; B: from white radish; C: from Chinese cabbage and pears (white kimchi); D: from kohlrabi; E: from ground cucumbers; F: stuffed Chinese cabbage in brine (ingredients: see Materials and Methods).

**Table 4:** Content of total polyphenols and antioxidant activity of selected types of kimchi.

Type of kimchi^a^	Total polyphenols (mg catechin/100 g)	ABTS (*μ*mol Tx/g)	DPPH (*μ*mol Tx/g)
A	193.7 ± 5.0^f^	132.3 ± 4.2^f^	49.7 ± 0.8^f^
B	135.8 ± 7.4^d^	67.5 ± 1.8^c^	21.0 ± 1.9^c^
C	112.9 ± 6.7^c^	75.1 ± 2.2^d^	23.9 ± 0.7^d^
D	79.2 ± 2.5^b^	50.6 ± 1.6^a^	18.7 ± 0.7^a^
E	146.6 ± 3.9^e^	88.7 ± 1.9^e^	36.3 ± 1.2^e^
F	67.4 ± 6.7^a^	58.2 ± 1.5^b^	19.3 ± 0.9^b^

Values are presented as the mean value ± SD (*n* = 4), in fresh matter. Different letters in the same columns indicate significant differences (*p* < 0.05). ^a^Type of kimchi—A: from Chinese cabbage; B: from white radish; C: from Chinese cabbage and pears (white kimchi); D: from kohlrabi; E: from ground cucumbers; F: stuffed Chinese cabbage in brine (ingredients: see Materials and Methods).

**Table 5:** Content of total flavonoids, flavonols, and phenolic acids of selected types of kimchi.

Type of kimchi^a^	Flavonoids (mg catechin/100 g)	Flavonols (mg quercetin/100 g)	Phenolic acids (mg caffeic acid/100 g)
A	55.20 ± 1.67^d^	20.45 ± 1.52^d^	22.82 ± 1.34^e^
B	20.94 ± 0.97^a^	10.25 ± 1.23^b,a^	11.51 ± 1.11^a,b^
C	21.47 ± 0.87^a^	11.85 ± 0.38^b,c^	13.32 ± 0.39^c^
D	22.51 ± 0.92^a^	9.55 ± 0.45^a^	10.10 ± 0.45^a^
E	45.74 ± 1.33^c^	24.21 ± 0.55^e^	20.86 ± 0.69^d^
F	24.83 ± 1.02^b^	12.28 ± 1.12^c^	12.33 ± 1.22^b^

Values are presented as the mean value ± SD (*n* = 4), in fresh matter. Different letters in the same columns indicate significant differences (*p* < 0.05). ^a^Type of kimchi—A: from Chinese cabbage; B: from white radish; C: from Chinese cabbage and pears (white kimchi); D: from kohlrabi; E: from ground cucumbers; F: stuffed Chinese cabbage in brine (ingredients: see Materials and Methods).

**Table 6:** Color parameters of selected types of kimchi.

Type of kimchi^a^	*L*∗	*a*∗	*b*∗	*C*∗	*h^o^*
A	46.73 ± 4.47^b,c,d^	8.76 ± 0.37^a^	28.62 ± 1.42^c^	30.63 ± 1.42^c^	73.15 ± 2.28^c^
B	32.63 ± 2.29^a^	6.85 ± 1.18^a^	22.43 ± 2.39^a^	23.61 ± 2.87^a^	71.80 ± 2.55^b,c^
C	53.16 ± 3.66^c,d^	6.35 ± 0.89^a^	24.96 ± 2.67^a,b^	24.90 ± 3.14^a^	74.51 ± 1.22^c^
D	41.24 ± 1.42^b^	11.00 ± 0.94^b^	22.01 ± 1.81^a^	24.07 ± 2.70^a^	62.93 ± 2.69^a^
E	33.93 ± 7.47^a^	12.10 ± 7.47^b^	27.20 ± 4.23^b,c^	30.04 ± 4.52^b,c^	66.11 ± 9.06^a,b^
F	48.24 ± 2.42^b,c,d^	17.27 ± 2.08^c^	23.50 ± 3.36^a^	27.62 ± 2.13^a,b^	65.19 ± 7.76^a^

Values are presented as the mean value ± SD (*n* = 4). Different letters in the same columns indicate significant differences (*p* < 0.05). ^a^Type of kimchi—A: from Chinese cabbage; B: from white radish; C: from Chinese cabbage and pears (white kimchi); D: from kohlrabi; E: from ground cucumbers; F: stuffed Chinese cabbage in brine (ingredients: see Materials and Methods).

## Data Availability

All data used to support the findings of this study are included within the article.
